# Increased neuroinflammatory and arachidonic acid cascade markers, and reduced synaptic proteins, in brain of HIV-1 transgenic rats

**DOI:** 10.1186/1742-2094-9-19

**Published:** 2012-01-23

**Authors:** Jagadeesh Sridhara Rao, Hyung-Wook Kim, Matthew Kellom, Dede Greenstein, Mei Chen, Andrew David Kraft, Gaylia Jean Harry, Stanley Isaac Rapoport, Mireille Basselin

**Affiliations:** 1 Brain Physiology and Metabolism Section, National Institute on Aging, 9000 Rockville Pike, Bldg. 9, 1S-126, Bethesda, MD 20892, USA; 2 National Institute of Mental Health, National Institutes of Health, Bethesda, MD 20892, USA; 3 Laboratory of Toxicology and Pharmacology, National Institute of Environmental Health Sciences, National Institutes of Health, Research Triangle Park, Durham, NC 27709, USA

## Abstract

Correction to Rao J S, Kim H W, Kellom M, Greenstein D, Chen M, Kraft A D, Harry G J, Rapoport S I, Basselin M. Increased neuroinflammatory and arachidonic acid cascade markers, and reduced synaptic proteins, in brain of HIV-1 transgenic rats. *Journal of Neuroinflammation *8:101.

## Correction

The authors observe that the original study [1] contains errors in the molecular weights for Figures 1, 2 and 3. The correct molecular weight for IL-1 beta should be 17 KD, TNF alpha- 17 KD, sPLA2-14 KD, 5-LOX 78 KD, 15-LOX 75 KD mPGES 42 KD and cytochrome p450 epoxygenase 56 KD.
